# The nexus of vitamin homeostasis and DNA synthesis and modification in mammalian brain

**DOI:** 10.1186/1756-6606-7-3

**Published:** 2014-01-10

**Authors:** Reynold Spector, Conrad E Johanson

**Affiliations:** 1 Robert Wood Johnson Medical School, 105 Stone Hill Road, Colts Neck NJ 07722, USA; 2 Department of Neurosurgery, Alpert Medical School at Brown University, 593 Eddy Street, Providence, RI 02903, USA

**Keywords:** Ascorbic acid, Folates, Hydroxymethyldeoxycytidine, Neuronal DNA, Ten-eleven translocation enzymes, Choroid plexus

## Abstract

The purpose of this review is to discuss the implications of the 2009 discovery of the sixth deoxyribonucleoside (dN) [5-hydroxymethyldeoxycytidine (hmdC)] in DNA which is the most abundant in neurons. The concurrent discovery of the three ten-eleven translocation enzymes (TET) which not only synthesize but also oxidize hmdC in DNA, prior to glycosylase removal and base excision repair, helps explain many heretofore unexplained phenomena in brain including: 1) the high concentration of ascorbic acid (AA) in neurons since AA is a cofactor for the TET enzymes, 2) the requirement for reduced folates and the dN synthetic enzymes in brain, 3) continued DNA synthesis in non-dividing neurons to repair the dynamic formation/removal of hmdC, and 4) the heretofore unexplained mechanism to remove 5-methyldeoxycytidine, the fifth nucleoside, from DNA. In these processes, we also describe the important role of choroid plexus and CSF in supporting vitamin homeostasis in brain: especially for AA and folates, for hmdC synthesis and removal, and methylated deoxycytidine (mdC) removal from DNA in brain. The nexus linking AA and folates to methylation, hydroxymethylation, and demethylation of DNA is pivotal to understanding not only brain development but also the subsequent function.

## Introduction

For over forty years, we and myriad others have investigated fluid (CSF) production by choroid plexus (CP) [1-3], ion and pH homeostasis in CSF [4-6], vitamin transport and homeostasis in CSF and brain [7-11], and DNA precursor transport/synthesis as well as DNA synthesis in developing and adult brain [12,13]. The importance of pH and ionic stability in CSF [14,15] and the extracellular space (ECS) of brain is immediately apparent: neurons cannot function properly with perturbations of pH and ions in CSF and brain ECS [16,17].

The transport and homeostatic mechanisms for certain vitamins (e.g., ascorbic acid (AA) and folates, subjects of this review), however, were unusual and unexpected [7,8,18]. In fact, for these two vitamins, which are concentrated ~ four times in both animal and human CSF, the one-hundred-year-old notion of the CSF as “nourishing liquor” for the brain turned out to be correct [8,18]. The mechanism for this physiologic process (with AA) was first illustrated by Hammarstrom [19] who demonstrated auto-radiographically that ^14^C-AA is transported from blood into CP, then CSF, next the brain adjacent to the CSF and finally, slowly, into the deep substance of the brain [8,19-22]. This route was subsequently also shown to be the way by which folates enter brain [23]. At that time we hypothesized that AA and folates enter brain predominantly via this circuitous route rather than directly via the blood–brain barrier (BBB) because this is an ontogenetic carry over. See below [23].

Moreover, from the CSF and ECF of brain, neurons concentrated AA to ~200 times the concentration of blood (~10 mM versus ~50 μM in plasma). Why do neurons require so much AA [20,24]? This puzzle has persisted for decades but, as described below, we now have a convincing although tentative answer. Similarly, why is there so much folate, and the enzymes to make the various folate cofactors in brain, e.g., for DNA synthesis [25,26]? There is ~25 times more folate in brain than plasma (0.02 μM). Again, an answer is now presented.

Yet another puzzle in both adult and aged mammals and humans, was that there are comprehensive transport systems and synthetic enzymes to ensure adequate and constant supplies of the precursors for active and ongoing widespread DNA synthesis in situ in adult and aged brain [13]. We wondered why, especially in adult human brain where there is no neuronal cell division except a 2% annual turnover in the dentate gyrus, would there be such a requirement for DNA synthesis [27]. Could it just be for routine DNA repair or mitochondrial duplication? This seemed an inadequate explanation. However, the discovery of ten-eleven translocation enzymes (TET) enzymes and 5-hydroxymethyldeoxycytidine (hmdC) in brain provides a partial answer as described below.

The purpose of this review is first to summarize briefly the transport and homeostatic systems for AA and folates in CSF and brain, and second the nature of the DNA precursor transport and enzyme systems in CP and brain. Following those comments, we then review the relevant findings and implications, beginning in 2009, of the discovery of the sixth deoxyribonucleoside (dN) in DNA, hydroxymethyldeoxycytidine (hmdC) [28,29]. We also recapitulate the data showing more hmdC in neurons (e.g., 0.6% of deoxynucleosides in nuclear DNA in Purkinje cells and neurons in general) than in any other cell-type [28]. Moreover, the amount of hmdC in brain neurons increases with age [30]. We will also recount the roles of hmdC in DNA as both a fairly stable marker [like methylated deoxycytidine (mdC) which makes up ~0.9% of the dN in DNA] and an unstable regulatory molecule modulating and controlling transcription of hundreds of genes; and how the discovery of hmdC and its removal, at last, explains the mechanism by which mdC can be removed from DNA [30-34]. Finally, we will assess the implications of the finding in 2013 that AA is a cofactor for the three ten-eleven translocation (TET) enzymes (TET 1–3) that produce and then, in some cases, oxidize hmdC in DNA prior to its removal, most likely by thymine DNA glycosylase (TDG) or possibly dC deaminase [34-36]. Subsequent to the removal of the oxidized hmdC from DNA, the DNA requires repair by the base excision repair (BER) mechanism in brain cells, a mechanism that requires the apparatus for DNA synthesis [31,35,36].

With this new information, we put forward an explanation for why hmdC in neurons helps explain the necessity for the high concentrations of AA, folate and the extensive machinery for DNA synthesis in adult and aged brain. Moreover, these findings have enormous implications for brain development and maturation as well as embryonic and adult stem cell research and cancer [34,35].

In order to preclude inappropriate conclusions, we will be very cautious about placing too much emphasis on tissue culture and other in vitro results, especially those done without AA in the medium. For example, Qiao and May in an elegant study [37] confirmed that in vivo human brain endothelial cells do not contain the sodium-dependent vitamin C transporter-2 (termed SVCT-2), the principal transporter of AA in CP and neurons [38] (see below). But as the endothelial cells divide in vitro, they acquire this transporter as do astrocytes which also do not express SVCT-2 in vivo. Thus, to paraphrase Song and He [34]: it is crucial to show that events that occur in vitro, e.g., in tissue culture, “indeed happen in vivo”. Their words were prophetic since some recent work was not conducted with AA in the medium for full TET activity; thus the formation/removal of hmdC in DNA was incomplete and many results that they and others have reviewed require reconsideration as discussed below.

### Ascorbic acid homeostasis in brain

Fifty years ago, Hughes showed that in AA-deficient guinea pigs, which like primates cannot synthesize AA, the brain was the last organ to be depleted of AA [20,21,39,40]. We now know that this is not due to lack of AA turnover. This finding helped explain why in severe human scurvy, CNS function is maintained as Lind pointed out centuries ago. As noted above, Hammarstrom [19] reported that AA was transported into brain via the CP and CSF [20,21]. In humans the CP is an organ weighing about 2 grams and receives blood flow at 4 to 5 ml/min/g. The CP epithelial cells, along with the arachnoid membrane, are the sites of the blood-CSF barrier [10,41,42]. Like the cerebral endothelial cells, the CP epithelial cells (not its capillaries) are joined by restrictive tight junctions [43]. Choroidal tight junctions are the anatomic substrate of the blood-CSF barrier (BCSFB) that restricts diffusion of molecules as small as urea [43]. Therefore, specialized membrane carriers are needed to move micronutrients (like vitamins), peptides and ions from blood into CSF [10,41].

Once in CSF, substances can penetrate the CSF-brain interfaces because there is a negligible barrier (gap junctions) to permeation via diffusion and pulsatile mixing from CSF into brain [42,44]. However, the process is slow. Some investigators have questioned whether substances can penetrate from CSF deep into the substance of the brain, but we, following Hammarstrom, have shown in rabbits that AA (m.w. 176) and mannitol (m.w. 182) penetrate deeply into rabbit brain from injections into ventricular CSF [22]. Others have shown that the much larger inulin (m.w. ~5000) can also slowly penetrate from CSF deep into brain [45].

Following Hammarstrom, we confirmed and extended his work in rabbits with ^14^C-AA in vitro and in vivo [20,21]. In humans, there is also strong evidence for AA transport into brain via CP-CSF rather than brain capillaries. Subsequent elegant work showed that the key AA transport system in CP is the SVCT-2 [38] that is not present in astrocytes or cerebral capillaries [24,37,46,47]. The SVCT-2 transporter is present on the basal (blood) side of the CP and in neurons [46,47]. In vivo, in mammals the CP pumps ~ one-half of the AA in plasma flowing through the CP into CSF with a step-up in concentration of ~4 times [48]. In neurons, AA is concentrated further by another ~50 times by membrane-bound SVCT-2. Thus, the concentration of AA in human plasma, CSF and neurons is ~50, 200 and 10,000 μM, respectively [24]. The validity of this model is corroborated by the phenotype of SVCT-2 knockout (KO) mice [49].

For details of AA systems in the CNS, there are recent reviews by us [8] and others, including a discussion of AA transport into and release from astrocytes by different mechanisms [24,46,47,50,51]. Still unknown is how AA exits the CP into CSF. However, what is clear is that the CNS homeostatic system is optimally developed to minimize fluctuation of the AA concentration in neurons [50]. Thus, at low plasma concentrations, relatively more AA is pumped into CSF via the CP; at high concentrations, relatively less, because the K_T_ (half saturation concentration) of SVCT-2 is close to the plasma concentration (~50 μM) [8,50]. Moreover, because the CSF and ECS of brain concentrations of AA are ~4 times higher than the plasma concentration, the SVCT-2 system in neurons is “saturated” except in extreme deficiency states [47,50].

Our explanation for the location of the SVCT-2 system in CP is that it is a carryover from early development when the CP is almost as big as the brain itself, presumably supplying nutrients to the latter [23]. However, as the forebrain grew in size, macronutrients like glucose and amino acids required the development of specialized carriers like glut-1 at the capillaries of the BBB. However, a few functions like micronutrient transport (e.g., AA and folate) were retained in CP [23]. Also, high levels of AA and folate bathing the subventricular zone where many neurons originate during development seem sensible. In elderly humans and Alzheimer’s disease patients, these CP transport functions remain normal [48].

Many investigators have offered reasons for the high concentration of AA in neurons, e.g., to serve as a cofactor for dopamine-β-hydroxylase which requires a millimolar concentration of AA for activity, for amidation of peptides, and for the synthesis of mature collagen [52]. However, these processes only occur in a small number of neurons (or other cells). Others have speculated that AA acts as a general antioxidant. In her excellent review, like us, Rice concluded that we do not know why there is so much AA in neurons [24].

Finally, in experiments analogous to the experiments with the TET enzymes described below, we studied a model of sympathetic neurons that synthesize norepinephrine (NE) from dopamine (DA) by DA-β-hydroxylase, an enzyme that requires millimolar concentrations of AA as a cofactor [52,53]. The model consisted of pheochromocytoma cells grown in tissue culture stimulated with nerve growth factor [53]. These cells then develop the properties of sympathetic neurons, e.g., extend varicose neurites that contain synaptic vesicles. If AA was added to the culture medium, NE was synthesized; if AA was left out, *minimal* NE was synthesized. In vitro, these cells take up AA by an active, saturable transport system. Moreover, the K_T_ for AA uptake into the differentiated cells was 36 μM. In retrospect, the AA carrier in these cells was almost certainly SVCT-2, the function of which was to concentrate intracellular AA to millimolar levels so that DA-β-hydroxylase could synthesize NE from DA (for relevance, see below).

### Folate transport into brain via CSF

The folate transport system in CP works similarly to the AA system, actively pumping methyltetrahydrofolate (MeTHF), the principal folate in plasma and CSF, from plasma into CSF [8,18,25,26,54]. Consequently, the steady-state concentration of MeTHF in CSF is ~2-4 times higher than plasma where ~50-60% of MeTHF is loosely bound to albumin [55]. In our early studies of the transport of folate, we discovered and purified the folate receptor α (FRα) in CP (pigs and humans) that binds both reduced and oxidized folates (e.g., folic acid) very tightly [54,56]. Our initial model of FRα, picking up MeTHF from blood (on the basal side of CP) and releasing it by facilitated diffusion on the apical side, was obviously incomplete [23]. How was the tightly-bound MeTHF released from the FRα [54,56]? This conundrum was solved in 2006 by Goldman’s group who discovered, cloned and characterized the proton-coupled folate transporter (PCFT; SLC 46A1) in gut and CP [57,58]. Presently the current model has CP transporting MeTHF from plasma into CSF, sequentially, from the vascular side of CP via membrane-bound FRα that becomes entrapped in pinched-off intracellular vesicles [18,57]. Then, PCFT releases the MeTHF from the vesicles into CP epithelial cytoplasm at pH ~7.1 [59]. Finally, by facilitated diffusion the MeTHF is released into CSF by the reduced folate carrier (RFC; SLC 19A1) at the apical side [60].

Several groups have confirmed and extended these animal findings to human CP and CSF [54,57]. Noteworthy is that the FRα is absent in cerebral capillaries [61]. Consequently, negligible MeTHF enters brain directly from blood [62]. Consistent with this model is that human KO’s of either FRα or PCFT manifest central folate deficiency (CFD) states, characterized by very low CSF folate concentrations and severe neurological disease or death [63-65]. Early treatment with mega-doses of reduced folates ameliorates CFD, however, and when started early enough, prevents CNS damage [64]. Evidently sufficient folate enters the brain to overcome the FRα or PCFT deficiency.

An acquired CFD, due to antibodies against FRα, can also be partially overcome by mega-doses of reduced folates [66]. Such experimental and clinical findings establish beyond doubt (in humans) the primary role of the CP in pumping folate from blood into CSF. Although controversy exists over the exact localization of FRα and PCFT in CP, however, it is clear that MeTHF is transported from plasma into CSF via CP [67,68]. Moreover, FRα-KO mice are not viable [65].

The exact mechanism(s) by which MeTHF enters neurons from CSF is unclear. Such concentrative uptake presumably involves the RFC present in neurons. In rabbits, however, unlike AA that turns over in brain relatively rapidly (t_½_ ~1 day), the folate turnover is exceedingly slow. Our best estimate in rabbits is a 7–10 day half-life [25,26]. A very slow turnover makes the study of brain MeTHF uptake more challenging.

Recently one group [67] postulated vesicular transport of MeTHF in FRα-containing vesicles released directly from CP into CSF, and then transported transependymally into brain. We think that this model needs substantiation for several reasons: 1) in earlier studies of ^14^C-MeTHF transport from blood to CSF, >73% of all untreated CSF samples yielded free ^14^C- MeTHF in column effluent; 2) untreated human CSF specimens emerge from HPLC columns as free MeTHF, a phenomenon not expected with vesicle-bound MeTHF [69]; 3) the FRα-containing vesicles were obtained from immortalized CP cells grown in culture, which as noted above, are prone to phenomena not occurring in vivo; 4) the authors do not explain how vesicles travel deep into the brain; and finally 5) the investigators do not cite the work of Holm et al. (1991) who extended/confirmed our work with human CP and CSF [54], demonstrating soluble FRα in human CSF (1 nM). Normally human CSF contains >40 nM MeTHF. For these reasons, unless the proponents of CSF vesicular transport provide definitive evidence that CSF, especially human, contains significant MeTHF in FRα-containing vesicles and that previous related research is incorrect, it appears that the unbound MeTHF diffusion/convection model for CSF described by several investigators [18,57] is currently the best fit of the collective data.

It is unequivocal that MeTHF is transported via CP from plasma into CSF by a system ~1/2 saturated at the normal plasma folate of ~20 nM [25,26]. Also, there is concurrence about the difficulty in depleting the healthy brain and CSF (with a normal CP) of folates. Although efficient and stabilizing, the brain homeostatic system for folates can be overcome by an extreme folate deficiency state or by a lack of FRα or PCFT in CP.

### The origin of DNA precursor molecules in adult brain

A) Role of transport from blood to central nervous system

In mammals, especially humans, it is clear that deoxyribonucleoside (dN) triphosphate precursors are predominantly synthesized de novo or salvaged in situ in brain [13,70]. In human plasma, the mean concentrations of uridine, hypoxanthine and thymidine are 3.1, 0.6, and 0.2 μM, respectively; in human lumbar CSF, 2.3, 2.5 and 0.06 μM, respectively [70]. The following observations pertain to human plasma [70]: 1) there was no detectable (0.1 μM) thymine, cytosine, guanine, cytidine or guanosine; 2) the other dNs present in DNA were also not detectable (0.1 μM); and 3) adenine and adenosine were present inconsistently with means of 0.3 and 0.2 μM, respectively. Thus, although there are weak (facilitated diffusion) systems for nucleosides and hypoxanthine at the BBB, except for uridine there is not enough dN and rN in human plasma to supply brain requirements for DNA synthesis [13].

In regard to bidirectional transport underlying CSF homeostasis of nucleotides, it is worth noting that CP contains a powerful system for removing rN and dN from CSF in vivo. On the apical, CSF-facing side of CP is the CNT3 transporter (SLC28a3) that actively and promiscuously clears rN and dN from CSF; the rN and dN (transported into CP from CSF) are released at the basal (blood) side of CP by an equilibrative nucleoside transporter (ENT1) [13]. Moreover, the human CSF thymidine concentration is 1/10 that of rabbit and rat [70]. Thus, extrapolating thymidine (or bromodeoxyuridine) pharmacokinetics from animals to humans is difficult. For a comprehensive review of the origin of dN in brain, see [13].

B) In situ salvage/synthesis of dN and DNA in developing and mature brain

In early work, we were surprised that dihydrofolate reductase (DHR), an enzymatic reducer of oxidized folates and dihydrobiopterin, to their tetrahydro derivatives, was reported as absent in brain in view of the serious toxicity of intrathecal methotrexate, a potent inhibitor of DHR [71]. With careful study, we were able to show unequivocally in developing and adult rabbit, rat and human brains that DHR is widely active. Subsequently we studied (directly and indirectly) in developing/mature rabbits and/or rats the mitochondrial and cytoplasmic forms of the salvage enzymes: deoxycytidine (dC) kinase and thymidine kinase (TK) [13]. These kinases are active throughout brain – even in adult and old animals.

Two synthetic enzymes, ribonucleotide reductase (RR) and thymidylate synthetase [TS] (employing methylene-THF as a cofactor), were also analyzed [13]; again, these synthetic enzymes displayed activity even in adult CNS. To assess activity of thymidylate synthetase in vivo, we injected ^3^H-deoxyuridine (dU) into the cerebral ventricles of temporarily-anesthetized rabbits. The radio-labeled dU reached brain, where the ^3^H-dU was converted into ^3^H-DNA via ^3^H-thymidine. This established that both the salvage enzymes (i.e., TK, which converts dU to dU monophosphate) and TS were active in vivo even in older rabbits. TS activity in human brain explains the CNS toxicity of 5-fluorouracil, an inhibitor of TS. It is also fascinating that neurospheres in vitro require reduced folates and will not accept thymidine (and salvage of dT by TK) [13]. If this phenomenon occurs in vivo (i.e., the necessity of requiring thymidine in DNA via TS), it implies that parenterally-labeled thymidine (or BrdU) misses sites of DNA synthesis in vivo; see [13] for further discussion of this point. Moreover, the protective enzyme dUTPase, which breaks down dUTP (capable of being erroneously incorporated into DNA), was active in adult and old rabbit brains [72].

Particularly surprising was the substantial activity of these enzymes in adult and aged brain. In some cases, e.g., RR and dUTPase, the enzymes were easily detected in adult and older brain, but not in liver. Moreover, the amount of actual DNA synthesis diffusely present in brain (clearly not for DNA replication since adult human neurons do not generally divide except in dentate gyrus [27]), in both the mitochondrial and especially the nuclear fractions, was unexpected. ‘Why the case’ is discussed below.

In related studies of brain and liver, we measured the methionine synthetase (MS) that employs MeTHF and vitamin B-12 to convert homocysteine to methionine [73,74]. Although methionine in plasma fluxes into brain across cerebral capillaries, apparently the blood-borne supply is insufficient. We established that MS, occurring in all brain regions, actively converts homocysteine into methionine in vivo in un-anesthetized adult rabbits [74]. This is a critical reaction because methionine is converted to the S-adenosylmethionine (SAM) that serves as a methyl donor in myriad reactions – the one of greatest interest here is the methylation of dC in DNA by both de novo and maintenance DNA methylases. A great mystery in biology, both in brain and other tissues, still remained. How are the folate-dependent methyl groups (via SAM) as mdC in DNA removed? It was clear that occasionally they undergo removal. There were many candidate mechanisms but none had been established in vivo.

Finally, also surprising was the stability of rN, dN and base concentrations in plasma and CSF. In adult rabbits, after a four-day fast with only water, there was no change in mean CSF rN, dN and base concentrations [75]. In plasma, only uridine changed (a 35% decline). In human plasma, after a four-day diet of only 100 g/day of glucose, there was no change in dN, rN or bases (except uridine, which declined by 36%) [76]. In humans on high-protein diets, the plasma uridine increases slightly. Therefore, except for uridine in plasma of rabbits and humans, there are homeostatic transport mechanisms throughout the body to maintain a steady concentration of rN, dN and purine/pyrimidine base concentrations.

### The discovery and implications of the hmdC in DNA

In 1983, like many others, we hypothesized that human DNA contains “abnormal” bases especially in old age and Alzheimer’s disease. On a sabbatical, one of us (RS) investigated this question on aged and Alzheimer’s brains using the best available techniques at that time. Nothing new was found [77] although hmdC was known to be in phage DNA.

In 2009, two groups presented unequivocal evidence that hmdC is indeed present in mammalian DNA, especially in neurons [28,29]. Moreover, one of these groups discovered and described the enzymatic mechanisms that oxidize mdC to hmdC in nuclear DNA, specifically TET 1, 2, and 3 [29]. They showed both Fe^++^ and α-ketoglutarate (α-KG) are TET co-factors. AA also has a co-factoring reducing role, i.e., to regenerate Fe^++^ from the Fe^+++^ formed in the oxidation reaction.

Considerable further work demonstrated that hmdC could then be oxidized by TET enzymes to 5-formyl dC (fdC) and then 5-carboxyl dC (cadC) [31-33]. Moreover, fdC and probably cadC could be removed by TDG with subsequent DNA repair by BER, thus requiring the four DNA dN triphosphate precursors [34-36].

A large series of related studies revealed that hmdC was involved in development, cancer, and transcription regulation in brain and other tissues. Significantly, the conundrum about how methyl groups are removed from mdC in DNA was finally solved by the recognition that TET enzymes can oxidize mdC in DNA, which could then be removed [35,36].

An independent remarkable observation was that, when AA was added to the medium, certain differentiated cells (e.g., mammalian B cells) could convert to embryonic-like stem cells capable of forming viable embryos [78]. This could not be done without AA in the medium. These groups however did not understand the mechanism. The critical breakthrough came when two groups established that AA was a co-factor for the TET enzymes [35,36]. AA is required to reduce inactive Fe^+++^ (formed when hmdC is synthesized in DNA) back to active Fe^++^, with the formation of DHA (dehydro-AA). DHA can then be reduced back to AA. This finding is reminiscent of our observation on NGF-treated pheochromocytoma cells that required AA in the medium to synthesize norepinephrine by dopamine-β-hydroxylase, an enzyme similar to the TET enzymes that also require AA [53]. Although iso-ascorbic acid (isoAA), a widely used anti-oxidant in food, is equally effective as AA as a cofactor for dopamine-β-hydroxylase and the TET enzymes, the isoAA has only ~5% the affinity for SVCT-2 as AA and hence is not transported into CP or neurons effectively [21].

**Figure 1:**
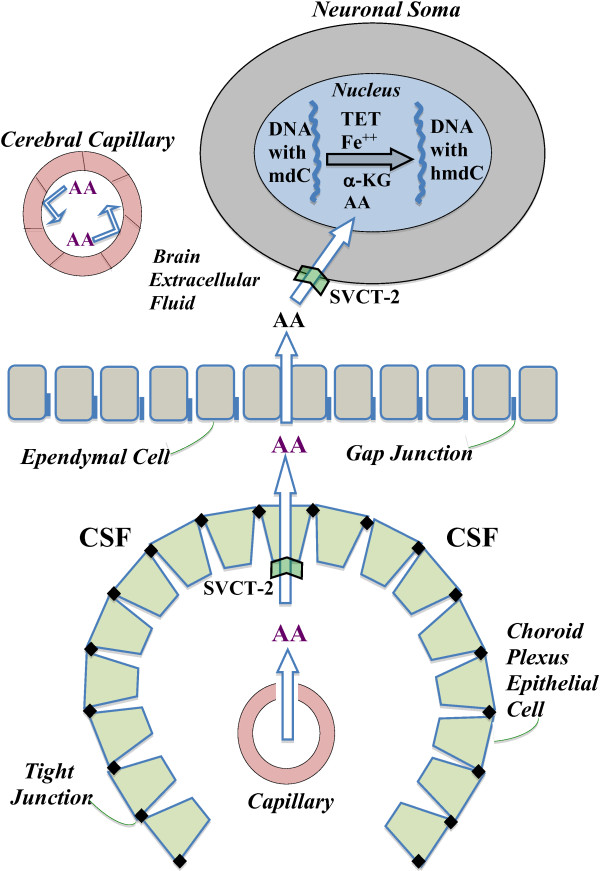
**Distributional route of ascorbic acid (AA) from blood into central nervous system to serve as a co-factor for TET enzymes in neuronal nuclei: Plasma-borne AA does *not *permeate the BBB, but is reflected off the inside surface of the capillary endothelium due to the restriction to diffusion offered by the tight junctions and luminal membrane (upper left).** Rather, AA reaches brain by a circuitous route involving (sequentially) the CP, CSF and ependyma. Thus, AA is transferred (unfilled arrow) from leaky blood vessels in CP through epithelial cells into ventricular CSF by the sodium-dependent vitamin C transporter 2 (SVCT-2) in the basolateral membrane. From CSF, AA diffuses freely through permeable gap junctions in the ependyma into brain extracellular fluid. There, AA reaches neurons and is greatly concentrated by the actively-transporting SVCT-2 in neuronal membranes. Upon accessing the neuronal cytoplasm, AA diffuses to and penetrates the nuclear membrane. Within the nucleoplasm, AA acts as an essential co-factor for TET (with Fe^++^ and α-KG) to oxidize certain methyldeoxycytidine (mdC) molecules in the same-strand DNA to hydroxymethyldeoxycytidine (hmdC). hmdC can be further oxidized and removed from DNA by thymine DNA glycosylase with subsequent DNA repair as explained in the text. The SVCT-2 transport systems for AA in CP and neurons act not only as concentrative systems for AA in CSF and neurons but also homeostatic systems that keep the millimolar concentration of AA in neurons relatively constant.

Finally, to assess the role of AA in vivo in brain, Yin et al. employed KO mice unable to synthesize AA [36]. They showed that compared to animals on an AA-supplemented diet, the mice on a severely-deficient diet (with ~50% as much AA in brain) had ~15% less hmdC (P<0.01) in their cerebral DNA. Given that there are ~12 million hmdC residues in diploid brain neuronal DNA, this suggests a decrease of ~2 million hmdC molecules per cell. Finally, there is now evidence that the TET enzymes and TDG are targets of micro RNA -29a, thus providing an ever more complex control of hmdC synthesis and removal in vivo [79]. It will take considerably more experimentation to understand the implications of hmdC in DNA, its control and the profound micronutrient effects on transcription phenomena. Figure 1 schematically depicts the access of AA to the neuronal nucleus, by a series of transport steps for AA from plasma to CP to CSF to brain extracellular fluid to uptake by the neuron and its nucleus; once in the nucleus, the AA (along with α-KG, Fe^++^ and TET) participates in the ongoing conversion of mdC to hmdC in the DNA strand.

### Implications and vistas

As described herein, we now have sound explanations for: 1) the need for active powerful systems in CP to concentrate AA and folates in CSF for ‘nourishment’ of neurons, 2) the high concentrations of AA and folates in neurons, and 3) the exquisite control of the CSF transport, salvage, and formation of the precursors of DNA synthesis in adult and aged mammalian brain, a tissue with relatively few dividing cells. As Minor et al. [35] postulated for cells such as neurons, with high levels of TET and hmdC in DNA, there would be a need for substantial concentrations of AA. This prescient suggestion was confirmed in vivo [36], as described above. Moreover, the explanation for the activation of hundreds of genes in cells grown in tissue culture with AA in the medium (versus controls with minimal AA) is now clear. Copious AA is required for optimal TET activity in the synthesis and removal of hmdC. Moreover, the removal of mdC would presumably activate many quiescent genes [35,36].

A second set of implications is tied to multiple effects of these findings on: the current methods such as DNA sequencing and “reading” of nuclear DNA (never mind the problem of ‘normal’ human and tumor mosaicism), the current conduct of genome-wide association studies that do not ‘dissect out’ mdC and hdmC in DNA, the use of certain restriction enzymes whose function in the face of hmdC needs investigation, and the ability of certain DNA-binding proteins and small RNA’s to bind to hmdC in DNA. This is just a smattering of questions and issues raised by the establishment of hmdC in DNA. Such queries are especially pertinent for neuronal DNA with its relatively large amount of hmdC.

A third implication is the challenge to the notion that in non-dividing cells, like neurons, the DNA sits in a relatively static state. This view is challenged by the finding of dynamic hmdC in DNA. The potential implications are profound: we suggest that these discoveries may lead to a better understanding of heretofore mysterious processes like memory and consciousness. Molecular tools are available to expedite the tremendous amount of work needed to obtain a full understanding of dynamic neuronal DNA. In a decade or two, the entire view of epigenetic physiology and neurogenetics could be revolutionized by the seminal findings of neuronal hmdC, the TET enzymes and the role of AA and folates as essential co-factors in brain nucleotide metabolism and DNA synthesis.

Finally, the work described in this explanatory review shows the inestimable value of anatomical, molecular, biochemical, physiological and pharmacological research of brain and CSF homeostatic systems. Almost certainly, this elucidation of brain physiology and cell biology will prompt incredible advances in the diagnosis and potentially epigenetic treatment of disease, including a better understanding of memory and even consciousness. The scientific door is now open and there is a clear and promising way forward.

## Abbreviations

α-KG: Alpha-ketoglutarate; AA: Ascorbic acid; BBB: Blood–brain barrier; BER: Base excision repair; cadC: 5-carboxyldeoxycytidine; CFD: Central folate deficiency; CP: Choroid plexus; DA: Dopamine; dC: Deoxycytidine; dN: Deoxyribonucleoside; dU: Deoxyuridine; dUTP: Deoxyuridine triphosphate; ECS: Extracellular space; fdC: 5-formyldeoxycytidine; FRα: Folate receptor alpha; hmdC: 5-hydroxymethyldeoxycytidine; isoAA: Iso-ascorbic acid; KO: Knock out; KT: Half saturation constant; mdC: Methylated deoxycytidine; MS: Methionine synthetase; NE: Norepinephrine; PCFT: Proton-coupled folate transporter; RFC: Reduced folate carrier; RR: Ribonucleotide reductase; SAM: S-adenosylmethionine; SVCT-2: Sodium-dependent vitamin C transporter-2; TDG: Thymine DNA glycosylase; THF: Tetrahydrofolate; TET: Ten-eleven translocation enzymes; TK: Thymidine kinase; TS: Thymidylate synthetase.

## Competing interest

The authors declare they have no competing interests.

## Authors' contributions

Both RS and CEJ analyzed the literature and wrote the manuscript. The authors carefully considered the BMC guidelines, and have read and approved the final manuscript.
